# Assessment of the Environmental Impact of Food Consumption in Ireland—Informing a Transition to Sustainable Diets

**DOI:** 10.3390/nu15040981

**Published:** 2023-02-16

**Authors:** Laura B. Kirwan, Janette Walton, Albert Flynn, Anne P. Nugent, John Kearney, Nicholas M. Holden, Breige A. McNulty

**Affiliations:** 1 School of Agriculture & Food Science, University College Dublin, Belfield, D04 V1W8 Dublin, Ireland; 2 Department of Biological Sciences, Munster Technological University, T12 P928 Cork, Ireland; 3 School of Food and Nutritional Sciences, University College Cork, T12 K8AF Cork, Ireland; 4 School of Biological Sciences, Institute for Global Food Security, Queens University Belfast, Belfast BT7 1NN, UK; 5 School of Biological & Health Sciences, Technological University Dublin, D07 EWV4 Dublin, Ireland; 6 School of Biosystems and Food Engineering, University College Dublin, Belfield, D04 V1W8 Dublin, Ireland

**Keywords:** sustainability, life cycle assessment, environment, environmental metrics, baseline, sustainable diets, food consumption, environmental impact, NDNS, national diet nutrition survey

## Abstract

Dietary changes are required to mitigate the climatic impact of food consumption. Food consumption databases can support the development of sustainable food based dietary guidelines (SFBDG) when linked to environmental indicators. An improved knowledge base is crucial to the transition to sustainable diets, and multiple environmental indicators should be considered to ensure this transition is evidence based and accounts for trade-offs. The current study aimed to quantify the environmental impact of daily diets across population groups in Ireland. Nationally representative food consumption surveys for Irish children (NCFSII; 2017–2018), teenagers (NTFSII; 2019–2020), and adults (NANS; 2008–2010) were used in this analysis. Blue water use (L) and greenhouse gas emissions (GHGe; kgCO_2_eq) were assigned at food level to all surveys. Cropland (m^2^), nitrogen (kgN/t), and phosphorous use (kgP/t) were assigned at the agricultural level for adults. Multiple linear regressions, Spearman correlations, and ANCOVAs with Bonferroni corrections were conducted. Higher environmental impact diets were significantly associated with demographic factors such as age, education status, residential location, and sex, but these associations were not consistent across population groups. The median greenhouse gas emissions were 2.77, 2.93, and 4.31 kgCO_2_eq, and freshwater use per day was 88, 144, and 307 L for children, teenagers, and adults, respectively. The environmental impact of the Irish population exceeded the planetary boundary for GHGe by at least 148% for all population groups, however the boundary for blue water use was not exceeded. Meat and meat alternatives (27–44%); eggs, dairy, and dairy alternatives (15–21%); and starchy staples (10–20%) were the main contributors to GHGe. For blue water use, the highest contributors were meat and meat alternatives in children; savouries, snacks, nuts, and seeds in teenagers; and eggs, dairy, and dairy alternatives in adults (29–52%). In adults, cropland use, nitrogen use, and phosphorous use exceeded planetary boundaries by 277–382%. Meat, dairy, and grains were the main contributors to cropland, nitrogen, and phosphorous use (79–88%). The quantified environmental impact of Irish diets provides a baseline analysis, against which it will be possible to track progress towards sustainable diets, and the basis for the development of Sustainable Food Based Dietary Guidelines in Ireland.

## 1. Introduction

The global food system is a major contributor to climate change [1], and the maintenance and protection of the environment, and managing uncertainty, are recognised as major challenges to meeting future global food demands [2]. The key environmental limits that encompass “planetary boundaries” refer to biodiversity loss; land use change; nitrogen cycling; phosphorous cycling; water use; and climate change resulting from greenhouse gas emissions (GHGe). If these boundaries are exceeded, ecosystems and related global regulatory processes are predicted to destabilise [1]. Worryingly, it is estimated that the safe operating space for climate change, land use change, and nitrogen and phosphorous cycling have already been exceeded at a European level [2]. Hence, maintaining the impact of food systems within the planetary boundaries is considered an important target in sustainable diet research and has already influenced international policy [3].

In Ireland, population level food based dietary guidelines (FBDG) were established in 2011, but adherence to these guidelines is not considered sufficient to meet environmental targets [4,5]. Alternatively, moving towards the EAT-LANCET diet is estimated to result in a 79% reduction in emissions in Ireland [5], yet the cultural acceptability, affordability, and nutritional adequacy of this diet has been questioned [6,7,8]. As existing FBDG are insufficient to meet international climate change targets, the development of sustainable food based dietary guidelines (SFBDG) has been recommended to mitigate the impact of global food systems [9]. For SFBDG to be effective, they need to consider baseline dietary patterns within the population and be established from a robust evidence base. The development of SFBDG has been hindered by the complex nature of quantifying environmental impact, and also due to many inconsistencies in data and methodologies [10]. Standardised indicators are thought to be underdeveloped at the food level for several environmental factors, and a high level of uncertainty is still recognised in this field [2,11,12]. Although environmental impact is considered multi-factorial, to date, relatively few factors have been considered in sustainable diet research. Typically research has focused on a single environmental metric, usually GHGe, which can be misleading and misrepresentative of the actual impact [10]. A lack of integrated analyses has resulted in the negligence of core environmental impact dimensions of food systems such as land use, water use, acidification, eutrophication, and carbon sequestration [10,13]. This may indicate a discord between science informing public health nutrition, and science informing climate action [10]. While momentum is building towards a more robust evidence base of sustainable diet research, a shared-knowledge framework has been suggested to direct more efficient future research, and the considerable uncertainties related to environmental factors need to be acknowledged [14].

Limited research to date has aimed to encompass the interconnections between food, health, and the environment. Nonetheless, a comprehensive overview of the interconnections between food, health, and the environment, with a “One Health” approach, recognising that the health of humans, animals, and the environment are linked and must be considered together was outlined by the Barilla Foundation. The “Double Pyramid” approach was developed, connecting food culture, health, and climate, and elaborates on the environmental impact of food production, the importance of considering local contexts, and the role of biodiversity in a healthy and sustainable food system [15]. Food labelling, consumer perceptions, dietary literacy, and food marketing should all be considered in the transition to sustainable diets [16].

For a transition to healthier and sustainable diets to be effective, an in-depth understanding of current dietary patterns, and their associated environmental impact, is required. Estimating and characterising the contribution of current dietary patterns to environmental impact may provide a scientific basis for designing SFBDG for Ireland [17]. The aim of the current study was to provide a more detailed assessment of the environmental impact of Irish diets across all population groups and to evaluate the relationship with environmental factors and nutritional adequacy.

## 2. Materials and Methods

### 2.1. Subjects and Data

Data on daily food intake at the individual level were acquired from three Irish national food consumption surveys: the National Adult Nutrition Survey (NANS; 2008–2010; 18–90 years), the National Teens Food Survey II (NTFSII; 2019–2020; 13–18 years), and the National Children’s Food Survey II (NCFSII; 2017–2018; 5–12 years) [18,19,20]. These food consumption databases (FCDB) were compiled from a series of nationally representative cross-sectional dietary surveys carried out by the Irish Universities Nutrition Alliance (IUNA)(www.iuna.net (accessed on 15 January 2023)). Ethical approvals for the IUNA surveys were obtained from the Clinical Research Ethics Committee of the Cork Teaching Hospitals and the Human Ethics Research Committee of University College Dublin for NANS [ECM 3 (p) 4 September 2008], NCFSII [ECM 4 (aa) 07/02/17], and NTFSII [ECM 4 (II) 04/12/2018]. In summary, the surveys assessed habitual food and beverage consumption and nutrient intakes using a four-day semi-weighed food record, in addition to compiling information on anthropometric measurements and physical activity [18,19,20]. Demographic factors such as age, gender, education level, residential location, and social economic status were collected across the surveys using a qualitative questionnaire [18]. Diets reported per day by participants were used in the current analysis, based on valid reported daily diets (energy intake to basal metabolic rate over 0.76) [21,22]. This resulted in 2375 reported diets for children, 798 diets for teenagers, and 4575 diets for adults. The study characteristics of the food consumption databases are available in Table S1. Further details of the survey methodologies are available at www.iuna.net (accessed on 15 January 2023).

### 2.2. Environmental Data Assigned to Foods as Consumed

GHGe and blue water use data were assigned to consumption data at food code level, using database values calculated using a life cycle assessment (LCA) that was previously mapped to the National Diet and Nutrition Survey (NDNS) in the UK [23]. Within this database, the blue (ground and surface water) water footprints (L/g food) of crop and livestock products were taken from data published by the Water Footprint Network (WFN) for the period 1996–2005 [24]. In the UK database, a weighted average was calculated based on the proportion of the total supply from various countries reported for both GHGe and water use, to account for the country of origin for imported foods [23]. Detailed information on the LCA database methodology, mapped to the Irish FCDB in the current study, is available elsewhere [23]. A validation check was completed to assess the relevance of the UK LCA database to the Irish market based on country of origins reported in the INFID database, with the foods largely produced and consumed from the UK/Ireland, and imported products, found to be consistent.

### 2.3. Environmental Data Assigned at the Agricultural Level

The Irish adult FCDB was previously converted from foods ‘as consumed’ to unprocessed foods at the agricultural level (wheat, rice, etc.) using the Irish Food Conversion Model (IFCM) [25]. Values for cropland, nitrogen, and phosphorous use were assigned to the adult food consumption data at the agricultural level only [26], as the food consumption databases for children and teenagers was not available as raw agricultural equivalents. In summary, data on cropland use were calculated using consumption data from the International Model for Policy Analysis of Agricultural Commodities and Trade (IMPACT) and data on primary production. Processed commodities were calculated using food conversion ratios and were split by economic value to avoid overlap. Country-level feed requirements were used to calculate cropland requirements for meat and dairy, and global feed requirements were used for aquaculture, with ratios adopted for wild and farmed fish production [26]. Data on fertiliser application rates were sourced from the International Fertilizer Industry Association [27] and were adjusted for efficiency gains from the rebalancing of fertiliser application rates between regions in line with closing yield gaps [28]. Detailed information on the FAOSTAT database is available online [26]. Additionally, GHGe were also assigned at the agricultural level to the Irish adult database (as recommended by the Intergovernmental Panel on Climate Change (IPCC) [29].

### 2.4. Planetary Boundaries and Environmental Impact Score

The planetary boundary approach assumes equal responsibility of all populations, and facilitates a comparison of the environmental impact of diets internationally [30,31]. Planetary boundaries were sourced from published literature and were calculated as the global food production-related mean planetary boundaries divided by the global population (1.87 kgCO_2_eq GHGe, 786 L freshwater use, 5.01 m^2^ land use, 27.4 gN, and 6.35 gP per capita per day) [8,31]. An environmental impact score was calculated by rescaling the five environmental factors to a score of 100, with equal weighting in adults. In children and teenagers, as only two environmental impacts were available, GHGe and water used were assessed separately for these groups and were not converted into an environmental score.

### 2.5. Statistical Analyses

Analyses were undertaken using the “R” statistical package version 4.1.1 (R Foundation for Statistical Computing, Vienna, Austria). Environmental factors were matched to appropriate codes, merged with food consumption files, multiplied by reported consumed amounts, and aggregated by food and demographic groups where appropriate. The environmental impact of diets is presented using elemental statistics including median output, interquartile range (25–75% IQR), and confidence intervals (5–95% CI). The relationship between environmental impact and additional factors was analysed using Spearman correlations and multiple linear regressions, with corrections for significant confounders (*p* < 0.05).

## 3. Results

### 3.1. Correlation between Environmental Factors, Food Weight, and Energy Intake

A significant positive relationship was found between energy (kcal/day) and food weight (g/day) intake for all environmental factors and population groups, and ranged from Rs 0.10 to 0.93. Energy density was calculated as (kcal from food per day)/(grams food weight per day). A significant negative relationship was also found between energy density and all environmental impacts in adults (Rs −0.09 to −0.61). Inter-correlations between environmental scores were assessed, with the weakest positive correlation found between GHGe and water use in adults (Rs 0.18, *p* < 0.001), whereas a particularly strong correlation was observed between cropland, nitrogen, and phosphorous use (Rs 0.99, *p* < 0.001) (Supplementary Table S2).

### 3.2. Population Level Results by Reported Daily Diets

**Figure 1:**
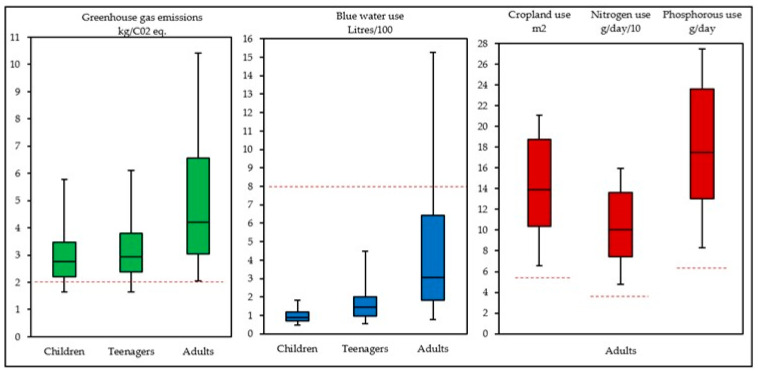
Environmental impact of diets reported in the Irish FCDB in relation to planetary boundaries (per capita). Cropland use is shown in m^2^; Nitrogen use in g/day/10; Phosphorous use is shown in g/day. Red dashed lines indicate planetary boundaries, calculated as food related limits divided by the global population [31]. Includes diets reported for children (*n* = 2375); teenagers (*n* = 798); and adults (*n* = 4575). Planetary boundaries as red broken lines as 1.87 kgCO_2_eq GHGe, 786 L blue water use, 5.01 m^2^ cropland use, 27.4 g N nitrogen use and 6.35 g P phosphorus use [32] per capita per day.

The environmental impact of each population group, based on the median reported daily diets, was first quantified, to assess the environmental impact relative to planetary boundaries. The median outputs were 2.77, 2.93, and 4.31 kgCO_2_eq for GHGe, and 88, 144, and 307 L blue water use per day for children, teenagers, and adults, respectively (Figure 1). When considering cropland, nitrogen, and phosphorous outputs based on the median reported diets for adults, an environmental impact of 14 m^2^, 100 kg N, and 17 kg P per day, respectively, was observed. In adults, to note, assigning GHGe values at the agricultural level resulted in a −1.27 and −0.67 kgCO_2_eq reduction in median values for male and female, respectively, resulting in a median GHGe output for adults of 3.30 ± 0.04 kgCO_2_eq per day. Planetary boundaries were exceeded by all population groups for GHGe, but not for water use, and adults exceeded the limits for cropland, nitrogen, and phosphorous use (Figure 1). The environmental impacts by gender and age groups are presented in more detail in supplementary materials Table S3 and Figures S1–S3.

### 3.3. Relationship between Environmental Impact and Demographic Factors

In children, higher GHGe outputs were associated with older children and those with parents with a lower education status. This was consistent for blue water usage, however, a significant relationship was also noted for those from an urban residential location. In teenagers, a significant association for GHGe was only found with male participants, whereas blue water use was significantly associated with older teenagers solely. In adults, younger participants, those with less skilled employment, males, and smokers were associated with higher GHGe outputs. For blue water use, higher outputs were associated with older participants, males, having a lower BMI, higher education, those in more skilled employment, those from a more rural residential location, and smokers. For cropland, nitrogen, and phosphorous use, a significant association was observed with younger adults, those in less skilled employment, males, and smokers across all three environmental impacts (Table 1).

### 3.4. Food Category Contributions to Environmental Impact

**Figure 2:**
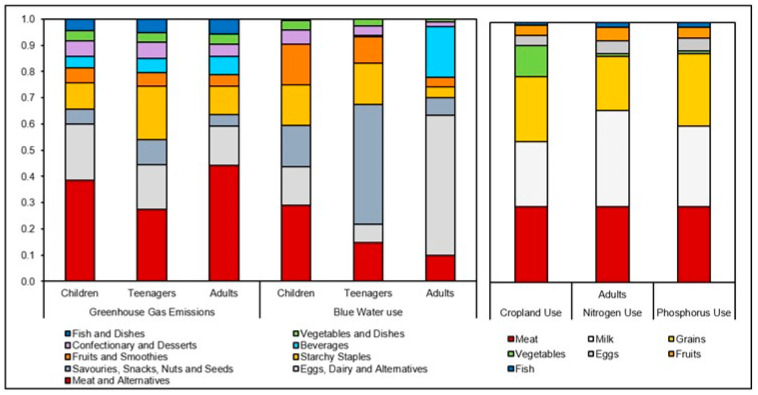
Percentage contribution of food categories to environmental impact for greenhouse gas emissions and blue water use (as food consumed) and for cropland, nitrogen and phosphorus use (as agricultural commodities). Values shown are median percentages across all three population groups for GHGe and blue water use, and for adults for the remaining factors. Includes diets reported for children (*n* = 2375); teenagers (*n* = 798); adults (*n* = 4575). Diets reported in the three surveys were weighted equally. Of the ‘eggs, dairy and alternatives’ group, dairy alternatives constitute 0.2%, 0%, and 0.2% for GHGe, and 6%, 0%, and 0.05% for children, teenagers, and adults for water use, respectively. Meat alternatives constitute 4%, 1%, and 3% for GHGe and 5%, 1%, and 1% for water use for children, teenagers, and adults, respectively. Starchy staples include breads and rolls; breakfast cereals; potatoes and products; and rice, pasta, flours, and starches.

To understand which food categories were driving each environmental factor, the contributions of nine overarching food categories were quantified and converted to percentages. An overview of the percentage contribution of each of these nine food categories to each environmental factor is presented in Figure 2. For GHGe, ‘meat and meat alternatives’ (27 –44%); ‘eggs, dairy, and dairy alternatives’ (15–21%); and ‘savouries, snacks, nuts, and seeds’ (10–20%) were the highest contributors across all population groups. For water use, similarly ‘savouries, snacks, nuts, and seeds’ (7–46%); ‘eggs, dairy, and dairy alternatives’ (7–53%); ‘meat and meat alternatives’ (10–29%); and ‘starchy staples’ (4–16%) were the highest contributors across all population groups. To note, over 60% of water output for teenagers and adults was attributed to the food categories: ‘eggs, dairy, and dairy alternatives’ (52%) and ‘beverages’ (19%) in adults; and ‘savouries, snacks, nuts and seeds’ (46%), and ‘starchy staples’ (15%) in teenagers. In children, the highest contributor to water use was ‘Meat and meat alternatives’, at 29%. For agricultural environmental impacts calculated for adults only, ‘meat and meat alternatives’ (29%); ‘eggs, dairy, and dairy alternatives’ (25–37%); and ‘rice, pasta, flours, and starches’ (21–28%) were the main contributors to cropland, nitrogen, and phosphorous use. Vegetables contributed 12% to cropland use, but only 1% to nitrogen and phosphorous use.

**Figure 3:**
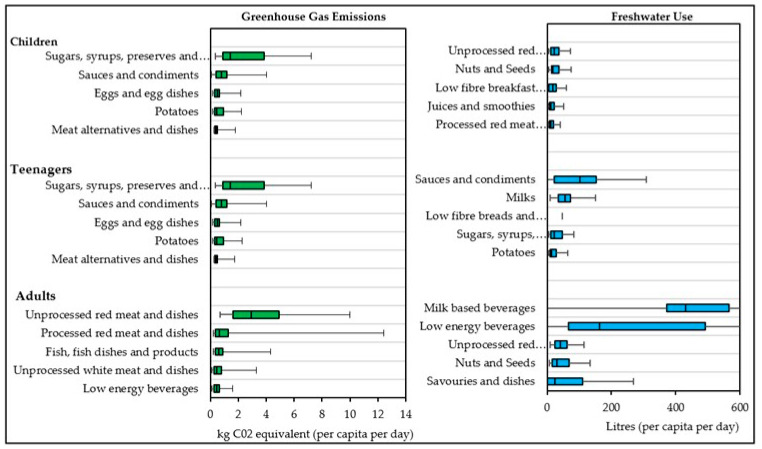
The main contributors to greenhouse gas emissions and blue water use across the population groups by actual intake amounts. Values shown as median, interquartile range (IQR) with 95% confidence intervals shown. Highest median contribution to each factor shown.

**Figure 4:**
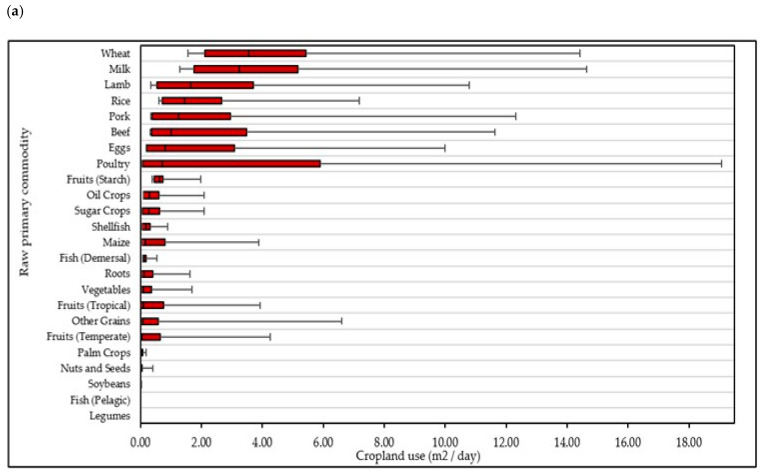

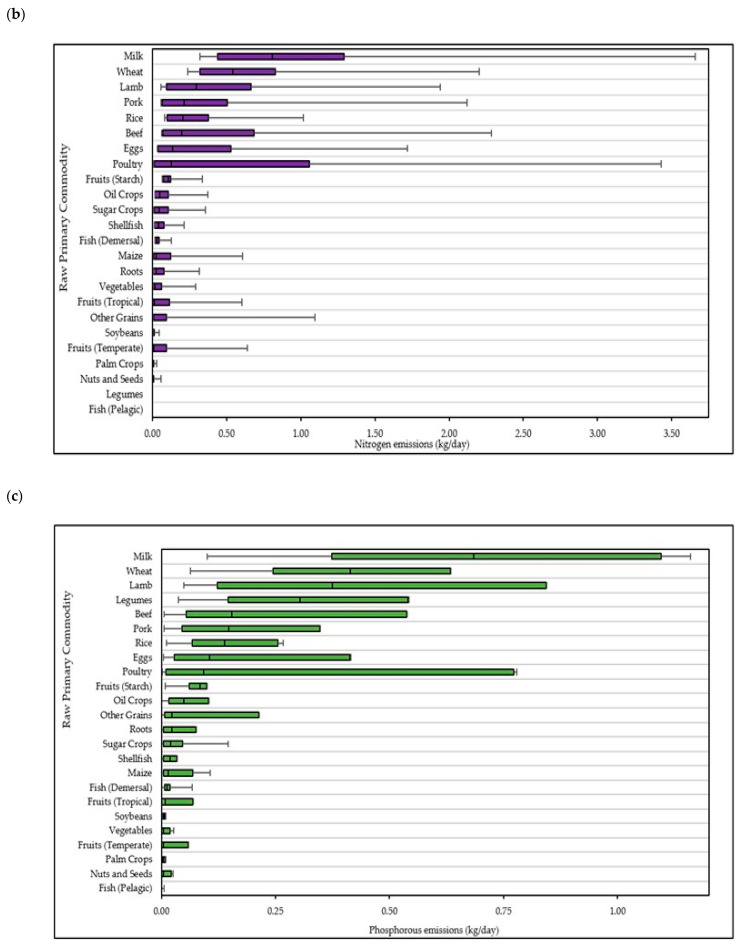
The contribution of foods to (**a**) cropland, (**b**) nitrogen, and (**c**) phosphorous use in Irish adults. Values shown as median, interquartile range (IQR) with 95% confidence intervals shown. Highest median contribution to each factor shown.

To examine food contributions at a detailed food level, the contribution of food categories to environmental impact was analysed using actual food weight values as opposed to percentage contributions (Figure 3). From this it was observed that, ‘sugars, syrups, preserves and sweeteners’; ‘sauces and condiments’; ‘eggs and egg dishes’; ‘potatoes’; and ‘meat alternatives and dishes’ were the highest contributors to GHGe in children and teenagers. In adults, ‘unprocessed red meat and dishes’; ‘processed red meats and dishes’, ‘fish, fish dishes and products’; ‘unprocessed white meat and dishes’; and ‘low energy beverages’ were the highest contributors to GHGe. For water use, ‘unprocessed red meat and dishes’; ‘nuts and seeds’; ‘low fibre breakfast cereals’; ‘juices and smoothies’; and ‘processed red meat and dishes’ were the highest contributors in children, whereas ‘sauces and condiments’; ‘milks’; ‘low fibre breads and rolls’; ‘sugars, syrups, preserves and sweeteners’; and ‘potatoes’ were the highest contributors in teenagers to blue water use. In adults, this was quite different again, with ‘milk-based beverages’; ‘low energy beverages’; ‘unprocessed red meats and dishes’; ‘nuts and seeds’; and ‘savouries and dishes’ being the highest contributors (Figure 3). In terms of cropland, nitrogen, and phosphorous use, ‘wheat’, ‘milk’, and ‘lamb’ were the top three contributing food categories (Figure 4). 

## 4. Discussion

This study is the first to quantify the environmental impact of food consumption in an Irish FCDB at a population sub-group level, and for multiple environmental factors. GHGe outputs were estimated at 2.77, 2.93, and 4.31 kgCO_2_eq for children, teenagers, and adults (per capita per day), respectively. For adults, this is lower than previous estimates of 6.5 kgCO_2_eq per day [33], which could be due to the use of a more recent environmental database, and a more detailed analysis. However, our GHGe estimations are more in line with the estimations made more recently based on baseline diet for an Irish adult, which had an output of 3.70 kgCO_2_eq per day for GHGe [34]. The study revealed that the reported diets in the Irish population are exceeding certain planetary boundaries. Diets exceeded the boundary for GHGe for all population groups; by 148% in children, 157% in teenagers, and 226% in adults. For adults, cropland, nitrogen, and phosphorous use also exceeded the planetary boundaries. Nonetheless, the boundary for blue water use was not exceeded by any population group. While meat and meat alternatives; dairy and alternatives; and starchy staples were recognised as the main contributors to environmental impact at an overarching level, when these were assessed at the food level, processed foods emerged as drivers of environmental impact for children and teenagers, and red meat for adults.

In a previous analysis on the environmental impact of a baseline diet of Irish adults (3.7 kgCO_2_eq, per day), the environmental impact was found to be approximately 0.5 kg per day higher than the average European reference diet (3.2 kgCO_2_eq) [34]. The main food categories (meat, dairy, and starchy staples) being high contributors to the environmental impact were consistent with a previous analysis in Irish adults [33], but the current study constitutes a more detailed and comprehensive environmental analysis. The TRUE project (TRansition paths to sUstainable legume-based systems in Europe) included an analysis of the environmental impact of food consumption in Irish adults based on average intakes, with an aim to identify the best routes, or “transition paths”, to increase sustainable legume cultivation and consumption across Europe. In the TRUE project, the contribution of meat, eggs, dairy and fish were estimated to account for 83% of Ireland’s GHGe [34], whereas here the estimate is 65% in Irish adults. However, when assessed at a more detailed food category level, the highest contributing foods were not consistent across population groups. In adults, ‘unprocessed red meat and dishes’ seemed to be driving GHGe, while in children and teenagers the highest contributing group was ‘sugars, syrups, preserves and sweeteners’, with ‘unprocessed red meat and dishes’ not featuring in the five contributors for either population group. The consistency between the high contributing food categories for both children and teenagers, and the difference with adults, may indicate a natural progression of dietary habits. These results also indicate the need for SFBDG which reflect the whole population’s dietary intakes, as the environmental impact in younger groups seems to be more associated with high intake energy-dense foods as opposed to animal sourced produce. Nevertheless, as the adult dietary data is older, the availability of updated data on adults would help to identify if these changes are due to changes over time or are related to the life stage of children and teenagers.

The correlations between food category intakes and total environmental impact also indicated differences between population groups, again with no single food category driving the environmental impact. It is possible that this may be attributable to energy over-consumption, as energy over-consumption was considerably higher in children and teenagers than in adults, with over 50% consuming energy in excess of their estimated requirements, whereas in adults this was only 20%. The higher contribution of these food categories may therefore relate to the amount being consumed as opposed to the environmental footprint of the food. Food policy may therefore need to target energy over-consumption, particularly in teenagers, where 40% of participants had energy intakes more than 180% of their energy requirements, as a strategy to reduce the environmental impact of food consumption. Further research correcting energy intakes may be required to indicate foods with higher environmental impacts, once over-consumption has been targeted. Nutritional adequacy is of critical importance when evaluating sustainable diets. The quantification of the environmental impact of diets in the current study to Irish FCDB, facilitates future analyses of environmental impact with additional factors, and future work should focus on analysing multiple components of a sustainable diet such as cost, nutritional adequacy, and environmental impact in tandem.

The relationship between demographic factors and environmental impact was also investigated. No demographic factor was found to be predictive of high or low environmental impact diets across all population groups and environmental factors assessed. Certain factors, such as sex, seem to play a role in individuals after childhood, whereby greater contributors were associated with being male. Parental education status was considered a significant factor for GHGe in children, but this was not apparent in teenagers or adults. In teenagers, this may be due to diminishing parental control over food choices as individuals become more independent [35]. The lack of an association of education level with GHGe is in line with findings of a study in the Netherlands, where higher education groups were found to have healthier diets, but no difference in environmental impact was found. This was surprising as the higher educated group had higher intakes of fruit (+28 g), vegetables (22+ g), and fish (6+ g) and a significantly lower intake of meat (men –33 g; women –14 g) than the lower education groups [36]. While children from an urban area were associated with increased blue water use, in comparison to children from a rural area, this was in contrast with adults, where a rural background was associated with higher water use. Residential location has also been found to influence food intakes and water use in a study in India [31]. Previous research has suggested dietary choice is dependent on the national food system, and that the environmental impact of food consumption needs to be completed at a population level and not extrapolated between regions [37]. The significant differences between population groups found in the current study suggest that even population level results are not sufficient, and context specific research is required.

While the quality and availability of environmental life cycle inventories (LCI) has improved, it is largely recognised that standardised guidance and databases are required to support a more robust and comparable analysis in the future. In an attempt to standardise environmental impact methods, the European Commission recently released guidance on quantifying and communicating the environmental impact of products [38]. In addition to this, the environmental impact of food consumption reported in the European Comprehensive Database has not yet been quantified, although a project was initiated in 2021 to develop an Environmental Footprint of Food (EFF) Database based on the Comprehensive European Food Consumption Database [39,40]. The FAO have also established a consensus building project to agree on the best practices for environmental and nutritional life cycle assessment (nLCA) methodologies, and to identify future research needs, with the first report released in 2021 [41]. While progress at a European and international level is therefore being made towards more standardised methodologies and data availability, standardised resources and methodologies to date remain limited for current research. Although not directly comparable, our results indicate a 28% lower mean and 44% lower median greenhouse gas emission output for adults when assigned at the agricultural level as opposed to ‘as consumed’, highlighting the impact the LCA methodology (assigning values at agricultural or product level) can have on outputs. While the current study quantifies the environmental impact of food consumption in Ireland, further research is required to elucidate an effective dietary strategy towards sustainable dietary habits in different population groups. The use of multi-dimensional modelling software could help to identify a transition to sustainable dietary patterns based on food combinations already being consumed in the population, and to identify synergies and trade-offs between factors. The Irish FCDB should be realigned with more progressive, harmonised, and standardised LCA methodologies, and on databases specific to Ireland, with respect to agricultural practices and production systems, when available. The dynamics between consumer choice, barriers, and behaviours relative to sustainability need to be investigated, and the clinical effects of altering diets to be sustainable need to be fully understood. The data behind the current study, when analysed with nutritional adequacy data, may provide a basis for the development of personalised dietary counselling towards healthier and sustainable diets.

The strengths of the current study include the use of detailed, high quality FCDBs and self-selected diets in the analysis. It also provides detailed analyses into multiple environmental factors across sub-groups in the Irish population, therefore providing context specific research, which was recently recommended to improve food systems research [11]. An evidence gap recognised in food systems research is a lack of detailed sub-group analyses [11]. The insight and data at the sub-group level of environmental outputs will be used for further research in sustainable diets in Ireland. Previous studies have been based predominantly on single benchmark diets, demand projections, and/or food balance sheets, which are not believed to be as accurate as food consumption data from individual-based dietary surveys [17,26]. Although environmental impact is multi-factorial, limited factors have been considered in sustainable diet research to date, with a single environmental metric, typically GHGe, assessed in isolation. This is considered misleading and may be misrepresentative of actual impact [10]. For example, in the current analysis, milk-based beverages were high contributors to freshwater use in adults, but not to GHGe. The use of self-selected, individual reported diets provides key data for identifying culturally accepted dietary patterns within a population and is useful for informing a transition to sustainable diets over time.

The limitations of the current study should also be noted. It is important to mention that reducing the environmental impact of a food system through dietary change is considered synergistic to measures relating to reduced food waste, improved agricultural practices and efficiency, and advancing technology in food production [10,42]. A large degree of uncertainty is recognised for all environmental data and sources used. The use of a single data point is not representative of the variability seen in agricultural practices. The use of average global figures does not incorporate agricultural practices from the country of production, particularly where FAO values were used for cropland, nitrogen, and phosphorus use, and more specific values would facilitate a more accurate analysis. While blue water use was considered, water scarcity was not accounted for. The planetary boundaries used in the current study were calculated based on global data from a previous publication [31] based on an EAT LANCET methodology [8]. This said, there are variations seen in planetary boundary calculations and results, and this should also be considered in the future. European planetary boundaries may provide more context specific insight into dietary impacts [32]. The food consumption data used for adults is from the period 2008–2010 and may not be fully representative of current diets in the population. In particular meat and dairy alternatives had very low consumption across all three surveys and were not substantially consumed. In the future, as these products become more widely available and consumption increases, these factors will have to be incorporated. It is, however, recognised that evidence is never complete, and a pragmatic approach using the available data has been recommended [11].

## 5. Conclusions

Food consumption databases constitute a valuable evidence base for the development of SFBDG. Current Irish diets are not sustainable, and exceeded all planetary boundaries apart from freshwater use. Higher environmental impact diets were significantly associated with demographic factors such as age, education status, residential location, and sex, but these were not consistent across population groups. This study fills a gap in the literature by assessing multiple indicators of environmental impact across a European population and provides a comprehensive and in-depth evaluation of the environmental impacts of food consumption. The insights from the current study provide a benchmark for tracking progress towards sustainable diets, international targets, and a basis for the development of SFBDG in Ireland across population groups. Further research should consider incorporating updated food consumption data on adults, more advanced data on environmental impacts, and additional factors such as dietary cost and nutritional adequacy.

## Figures and Tables

**Table 1:** Correlation between environmental impact and demographic factors.

	GHGe	Water Use	Cropland Use	Nitrogen	Phosphorus
	kgCO_2_eq	Litres	m^2^	gN	gP
Children					
Age	0.10 (0.07, 0.13) ***	6.06 (4.84, 7.27) ***	-	-	-
Sex	0.05 (−0.07, 0.16)	−6.77 (−11.85, −1.68) *	-	-	-
Social class †	0.00 (−0.07, 0.07)	2.00 (−0.99, 4.98)	-	-	-
Education †	−0.42 (−0.54, −0.31) ***	0.72 (−4.44, 5.87)	-	-	-
Residential location	0.03 (−0.02, 0.08)	4.86 (2.66, 7.06) ***	-	-	-
BMI	0.03 (0.01, 0.05) *	0.03 (−0.98, 1.05)	-	-	-
Teenagers					
Age	0.09 (−0.01, 0.18)	22.07 (12.93, 31.22) ***	-	-	-
Sex	−1.11 (−1.4, −0.82) ***	14.81 (−13.06, 42.69)	-	-	-
Education †	0.01 (−0.28, 0.31)	32.2 (3.87, 60.52).	-	-	-
Social class †	0.04 (−0.12, 0.19)	−10.91 (−25.76, 3.93)	-	-	-
Residential location	0.14 (−0.01, 0.29)	15.43 (0.91, 29.96).	-	-	-
BMI	0.02 (−0.03, 0.06)	0.83 (−3.64, 5.3)	-	-	-
Adults					
Age	−0.02 (−0.03, −0.01) ***	4.10 (3.22, 4.98) ***	−0.06 (−0.08, −0.05) ***	−0.46 (−0.56, −0.35) ***	−0.08 (−0.10, −0.06) ***
Sex	−2.46 (−2.67, −2.25) ***	−70.91 (−96.22, −45.6) ***	−3.16 (−3.59, −2.74) ***	−20.08 (−22.99, −17.16) ***	−3.53 (−4.03, −3.03) ***
Residential location	0.01 (−0.08, 0.10)	−28.10 (−38.51, −17.69) ***	0.03 (−0.15, 0.2)	0.00 (−1.20, 1.20)	0.00 (−0.21, 0.20)
Education	−0.06 (−0.19, 0.06)	47.49 (32.63, 62.36) ***	0.02 (−0.23, 0.27)	0.43 (−1.28, 2.15)	0.07 (−0.22, 0.37)
Employment	0.08 (0.02, 0.15) *	−21.69 (−29.46, −13.93) ***	0.20 (0.07, 0.33) *	1.30 (0.41, 2.20) *	0.23 (0.07, 0.38) *
Social class	0.09 (−0.02, 0.20)	−6.86 (−20.08, 6.36)	−0.09 (−0.31, 0.14)	−0.86 (−2.38, 0.67)	−0.14 (−0.41, 0.12)
BMI	0.17 (0.03, 0.31).	−26.00 (−42.97, −9.03) *	0.06 (−0.23, 0.34)	0.14 (−1.82, 2.10)	0.01 (−0.33, 0.34)
Ethnic group	−0.42 (−1.15, 0.31)	−12.64 (−99.8, 74.52)	0.80 (−0.67, 2.27)	4.69 (−5.36, 14.73)	0.79 (−0.94, 2.52)
Non-smoker	−0.28 (−0.41, −0.15) ***	−104. (−120.5, −88.93) ***	−0.55 (−0.81, −0.28) ***	−3.32 (−5.14, −1.5) **	−0.58 (−0.89, −0.26) **

Abbreviations: CO_2_ = carbon dioxide. m^2^ = metres squared. g = grams. n = nitrogen. p = phosphorous. BMI = body mass index. Multiple linear regression results corrected for significant factors. Values shown as β2 (5–95% confidence interval). Significance denoted at the *** *p* < 0.001 ** *p* < 0.01 * *p* < 0.05 levels “.” *p* < 1.0. Residential location was grouped across surveys as 1. Open country, 2. Small town (1500–9999 habitants), 3. Large town (10,000+ habitants), and 4. City. Social class coded as 1. Professional/managerial/technical, 2. Non-manual skilled, 3. Manual skilled, and 4. Semi-skilled/unskilled. † Indicates data recorded for participants’ guardian(s).

## Data Availability

The data presented in this study are available from the corresponding author upon reasonable request.

## Supplementary Materials

The following supporting information can be downloaded at: https://www.mdpi.com/article/10.3390/nu15040981/s1, Table S1: Demographic characteristics of food consumption databases when used as daily diets. Table S2: Correlation between environmental factors, total energy, and food weight. Table S3: Environmental impact of diets by age group and environmental factor. Figure S1: GHGe from daily reported diets in the food consumption surveys across population groups. Figure S2: Blue water use from daily reported diets in the food consumption surveys across population groups. Figure S3: Cropland, nitrogen, and phosphorus use from daily reported diets for adults.

## References

[1] Springmann M., Mason-D’Croz D., Robinson S., Garnett T., Godfray H.C.J., Gollin D., Rayner M., Ballon P., Scarborough P. (2016). Global and regional health effects of future food production under climate change: A modelling study. Lancet.

[2] Macintosh K.A., Chin J., Jacobs B., Cordell D., McDowell R., Butler P., Haygarth P., Williams P., Quinn J.P., O’Flaherty V. (2019). Transforming phosphorus use on the island of Ireland: A model for a sustainable system. Sci. Total Environ..

[3] FAO (2017). The Sustainable Development Goals Report 2017.

[4] Martini D., Tucci M., Bradfield J., Di Giorgio A., Marino M., Del Bo’ C., Porrini M., Riso P. (2021). Principles of Sustainable Healthy Diets in Worldwide Dietary Guidelines: Efforts So Far and Future Perspectives. Nutrients.

[5] Springmann M., Spajic L., Clark M.A., Poore J., Herforth A., Webb P., Rayner M., Scarborough P. (2020). The healthiness and sustainability of national and global food based dietary guidelines: Modelling study. BMJ.

[6] Drewnowski A. (2020). Analysing the affordability of the EAT–*Lancet* diet. Lancet Glob. Health.

[7] Lassen A.D., Christensen L.M., Trolle E. (2020). Development of a Danish Adapted Healthy Plant-Based Diet Based on the EAT-Lancet Reference Diet. Nutrients.

[8] Willett W., Rockström J., Loken B., Springmann M., Lang T., Vermeulen S., Garnett T., Tilman D., DeClerck F., Wood A. (2019). Food in the Anthropocene: The EAT–Lancet Commission on healthy diets from sustainable food systems. Lancet.

[9] FAO, WHO (2019). Sustainable Healthy Diets.

[10] Ridoutt B.G., Hendrie G.A., Noakes M. (2017). Dietary Strategies to Reduce Environmental Impact: A Critical Review of the Evidence Base. Adv. Nutr..

[11] Deconinck K., Giner C., Jackson L.A., Toyama L. (2021). Overcoming Evidence Gaps on Food Systems.

[12] Perignon M., Vieux F., Soler L.-G., Masset G., Darmon N. (2017). Improving diet sustainability through evolution of food choices: Review of epidemiological studies on the environmental impact of diets. Nutr. Rev..

[13] Grigoriadis V., Nugent A., Brereton P. (2021). Working towards a combined measure for describing environmental impact and nutritive value of foods: A review. Trends Food Sci. Technol..

[14] Vieux F., Privet L., Soler L., Irz X., Ferrari M., Sette S., Raulio S., Tapanainen H., Hoffmann R., Surry Y. (2020). More sustainable European diets based on self-selection do not require exclusion of entire categories of food. J. Clean. Prod..

[15] Barilla Foundation & Research Unit on Nutrition, Diabetes and Metabolism, University of Naples Federico II (2021). A One Health Approach to Food, the Double Pyramid Connecting Food Culture, Health and Climate.

[16] Shangguan S., Afshin A., Shulkin M., Ma W., Marsden D., Smith J., Saheb-Kashaf M., Shi P., Micha R., Imamura F. (2019). A Meta-Analysis of Food Labeling Effects on Consumer Diet Behaviors and Industry Practices. Am. J. Prev. Med..

[17] Del Gobbo L.C., Khatibzadeh S., Imamura F., Micha R., Shi P., Smith M., Myers S.S., Mozaffarian D. (2015). Assessing global dietary habits: A comparison of national estimates from the FAO and the Global Dietary Database. Am. J. Clin. Nutr..

[18] IUNA (2011). National Adult Nutrition Survey (NANS) Summary Report on Food and Nutrient Intakes, Physical Measurements, Physical Activity Patterns and Food Choice Motives.

[19] IUNA, National Children’s Food Survey II (NCFSII) (2018). Main Survey Report.

[20] IUNA, National Teen’s Food Survey II (NTFSII) (2020). Main Survey Report.

[21] Schofield W.N. (1985). Predicting basal metabolic rate, new standards and review of previous work. Hum. Nutr. Clin. Nutr..

[22] Goldberg G.R., Black A.E., Jebb S.A., Cole T.J., Murgatroyd P.R., Coward W.A., Prentice A.M. (1991). Critical evaluation of energy intake data using fundamental principles of energy physiology: 1. Derivation of cut-off limits to identify under-recording. Eur. J. Clin. Nutr..

[23] Scheelbeek P., Green R., Papier K., Knuppel A., Alae-Carew C., Balkwill A., Key T.J., Beral V., Dangour A.D. (2020). Health impacts and environmental footprints of diets that meet the Eatwell Guide recommendations: Analyses of multiple UK studies. BMJ Open.

[24] Mekonnen M.M., Hoekstra A.Y. (2011). The green, blue and grey water footprint of crops and derived crop products. Hydrol. Earth Syst. Sci..

[25] DAFM (2017). Disaggregation of Food Consumption Databases to Raw Agricultural Commodity Values for Estimation of Intakes of Pesticide Residues RACConvert Final Report.

[26] Springmann M. (2018). Health and nutritional aspects of sustainable diet strategies and their association with environmental impacts: A global modelling analysis with country-level detail. Lancet Planet. Health.

[27] International Fertilizer Association (IFA), International Plant Nutrition Institute (IPNI) (2017). Assessment of Fertilizer Use by Crop at the Global Level 2014-2014/15.

[28] Mueller N.D., Gerber J.S., Johnston M., Ray D.K., Ramankutty N., Foley J.A. (2012). Closing yield gaps through nutrient and water management. Nature.

[29] IPCC (2019). Climate Change and Land: An IPCC Special Report on Climate Change, Deforestation, Sustainable Land Management, Food Security, and Greenhouse Gas Fluxes in Terrestrial Ecosystems at its 50th Session Held on 2–7 August 2019.

[30] Moberg E., Potter H.K., Wood A., Hansson P.-A., Röös E. (2020). Benchmarking the Swedish Diet Relative to Global and National Environmental Targets—Identification of Indicator Limitations and Data Gaps. Sustainability.

[31] Chaudhary A., Krishna V. (2021). Region-specific nutritious, environmentally friendly, and affordable diets in India. One Earth.

[32] European Environment Agency (EEA) (2020). Is Europe Living within the Limits of Our Planet? An Assessment of Europe’s Environmental Footprints in Relation to Planetary Boundaries.

[33] Hyland J.J., Henchion M., McCarthy M., McCarthy S.N. (2017). The climatic impact of food consumption in a representative sample of Irish adults and implications for food and nutrition policy. Public Health Nutr..

[34] Williams M., O’Driscoll B., Saget S., Iannetta P., Styles D. (2020). A Combined Environmental and Nutri-Economic Assessment of Diets. Deliverable (D) 5.6 (D34) for the EU-H2020 Project. Transition Paths to sUstainable Legume-Based Systems in Europe (TRUE).

[35] Daly A.N., O’Sullivan E.J., Walton J., McNulty B.A., Kearney J.M. (2020). Eating behaviour styles in Irish teens: A cross-sectional study. Public Health Nutr..

[36] van Bussel L.M., Van Rossum C.T., Temme E.H., Boon P.E., Ocké M.C. (2020). Educational differences in healthy, environmentally sustainable and safe food consumption among adults in the Netherlands. Public Health Nutr..

[37] Mertens E., Kuijsten A., van Zanten H.H., Kaptijn G., Dofková M., Mistura L., D’Addezio L., Turrini A., Dubuisson C., Havard S. (2019). Dietary choices and environmental impact in four European countries. J. Clean. Prod..

[38] Directorate-General for Environment (2021). Recommendation on the Use of Environmental Footprint Methods.

[39] EFSA (2021). Creation of Open Access EU Food Composition Database (EU FCDB) and Related Datasets GP/EFSA/DATA/2021/02, in D01.01-DATA-29.

[40] EFSA (2021). The EFSA Comprehensive European Food Consumption Database. https://www.efsa.europa.eu/en/food-consumption/comprehensive-database.

[41] McLaren S., Berardy A., Henderson A., Holden N., Huppertz T., Jolliet O., De Camillis C., Renouf M., Rugani B., Saarinen M. (2021). Integration of Environment and Nutrition in Life Cycle Assessment of Food Items: Opportunities and Challenges.

[42] Ritchie H., Roser M. Environmental Impacts of Food Production. Published online at OurWorldInData.org. https://ourworldindata.org/environmental-impacts-of-food.

