# Supplementary material for: Assessment of the Environmental Impact of Food Consumption in Ireland—Informing a Transition to Sustainable Diets
Source: Nutrients. 2023 Feb 16;15(4):981. doi: 10.3390/nu15040981 (PMC9958966; doi:10.3390/nu15040981)
Supplement: Supplementary file 1 [file nutrients-15-00981-s001.zip › nutrients-2195371-supplementary.pdf]

Table S1. Demographic characteristics of population food consumption databases used as daily diets.

	Children	Teenagers	Adults
	n 2375	*n 798*	*n 4575*
Age group (years)			
5 - 8	1192 (50%)	-	-
9 - 12	1183 (50%)	-	-
13 - 15	-	445 (56%)	-
16 - 18	-	353 (44%)	-
18 - 35	-	-	1690 (37%)
36 - 50	-	-	1407 (31%)
51 - 64	-	-	910 (20%)
65 +	-	-	567 (12%)
Sex			
Female	1186 (50%)	389 (49%)	2340 (51%)
Male	1189 (50%)	409 (51%)	2234 (49%)
Education (%)			
Primary and intermediate	146 (6%)	40 (5%)	1230 (27%)
Secondary	175 (7%)	91 (11%)	1062 (23%)
Tertiary	2042 (86%)	662 (83%)	2234 (49%)
Location (%)			
Open country/village (≤1499)	945 (40%)	306 (38%)	1327 (29%)
Small town (1500 - 9999)	307 (13%)	276 (35%)	180 (4%)
Large town (≥10000)	685 (29%)	166 (21%)	1593 (35%)
City (Dublin/Cork)	438 (18%)	50 (6%)	1474 (32%)
Social class (%)			
Professional/managerial/technical	1573 (67%)	465 (60%)	2166 (49%)
Non manual workers	407 (17%)	123 (16%)	780 (18%)
Skilled manual workers	211 (9%)	113 (15%)	596 (13%)
Semi-skilled/unskilled/students	168 (7%)	77 (10%)	890 (20%)

Abbreviations: n = number of daily diets. Missing variables; Education (n12, 5 and 48); Social Class (n16, 20, 142) for children, teenagers, and adults respectively.

Table S2. Correlation between environmental factors, total energy, and food weight.

		**Blue water use**		**GHGe**		**Cropland use**		**Nitrogen use**		**Phosphorus use**	
		R_s	P	R_s	P	R_s	P	R_s	P	R_s	P
Children	Food weight (g/d)	0.36	<0.001	0.54	<0.001	-	-	-	-	-	-
	Total energy (kcal/d)	0.40	<0.001	0.58	<0.001	-	-	-	-	-	-
	Blue water use (L/day)	1.00	<0.001	0.51	<0.001	-	-	-	-	-	-
	GHGe (kg C02 eq/day)	0.51	<0.001	1.00	<0.001	-	-	-	-	-	-
Teenagers	Food weight (g/d)	0.23	<0.001	0.54	<0.001	-	-	-	-	-	-
	Total energy (kcal/d)	0.19	<0.001	0.54	<0.001	-	-	-	-	-	-
	Blue water use (L/day)	1.00	<0.001	0.32	<0.001	-	-	-	-	-	-
	GHGe (kg C02 eq/day)	0.32	<0.001	1.00	<0.001	-	-	-	-	-	-
Adults	Food weight (g/d)	0.23	<0.001	0.54	<0.001	0.93	<0.001	0.93	<0.001	0.93	<0.001
	Total energy (kcal/d)	0.10	<0.001	0.55	<0.001	0.43	<0.001	0.42	<0.001	0.43	<0.001
	Energy density (kcal/g)	-0.15	<0.001	-0.09	<0.001	-0.60	<0.001	-0.61	<0.001	-0.61	<0.001
	Blue water use (L/day)	1.00	<0.001	0.18	<0.001		<0.001	0.22	<0.001	0.22	<0.001
	Cropland use (m^2/day)	0.22	<0.001	0.50	<0.001	1.00	<0.001	0.99	<0.001	1.00	<0.001
	GHGe (kg C02 eq/day)	0.18	<0.001	1.00	<0.001	0.50	<0.001	0.47	<0.001	0.48	<0.001
	Nitrogen use (kg/day)	0.22	<0.001	0.47	<0.001	0.99	<0.001	1.00	<0.001	1.00	<0.001
	Phosphorus use (kg/day)	0.22	<0.001	0.48	<0.001	1.00	<0.001	1.00	<0.001	1.00	<0.001

Spearmann Correlations with P values <0.05 considered significant. Energy density is calculated as kcal from food/grams food weight per day. Abbreviations: g/d = grams per day. kcal/day = calories (energy) per day. L/day = litres per day. GHGe = greenhouse gas emissions. m^2/day = metres squared per day. kg = kilograms. C02 eq. = carbon dioxide equivalent. Rs = Spearmann correlation coefficient. P = value of significance.

Table S3. Environmental impact of diets by age group and environmental factor.

Age group	Environmental factor	Females			
		Mean ± SD	Median ± SEM	IQR	95% CI
5 - 8	Blue water use (L)	85.80 ± 41.40	76.30 ± 3.50	59.60 - 102.00	45.20 - 156.00
	GHGe (kgC02eq)	2.60 ± 0.89	2.50 ± 0.08	2.02 - 3.12	1.55 - 4.35
9 - 12	Blue water use (L)	115.0 ± 59.40	103.0± 5.80	75.90 - 140.00	57.80 - 211.00
	GHGe (kgC02eq)	2.90 ± 0.93	2.80 ± 0.09	2.39 - 3.25	1.89 - 4.33
13 - 15	Blue water use (L)	129.00 ± 113.00	81.30 ± 15.20	66.90 - 152.00	38.60 - 408.00
	GHGe (kgC02eq)	2.36 ± 1.07	2.10 ± 0.14	1.68 - 2.68	1.25 - 4.46
16 - 18	Blue water use (L)	221.00 ± 240.00	148.00 ± 33.20	89.50 - 230.00	41.40 - 685.00
	GHGe (kgC02eq)	2.30 ± 0.86	2.30 ± 0.12	1.77 - 2.74	1.30 - 3.91
18 - 35	Blue water use (L)	370.00 ± 395.00	223.00 ± 16.50	125.0 - 452.00	55.60 - 1285.00
	Cropland use (m^2)	15.20 ± 7.10	13.80 ± 0.29	10.20 - 18.40	6.62 - 30.20
	GHGe (kgC02eq)	4.80 ± 3.00	3.70 ± 0.13	2.88 - 5.66	2.12 - 11.50
	Agri. GHGe (kgC02eq)	2.82 ± 0.07	3.29 ± 1.90	1.94 - 4.09	1.31 - 7.02
	Nitrogen use (kg)	110.00 ± 50.10	101.00 ± 2.09	72.50 - 135.00	48.80 - 213.00
	Phosphorous use (kg)	19.10 ± 8.60	17.70 ± 0.36	12.70 - 23.20	8.50 - 36.70
36 - 50	Blue water use (L)	640.00 ± 522.00	468.00 ± 22.5	241.0 - 874.0	119.00 - 1636.00
	Cropland use (m^2)	15.40 ± 6.340	14.00 ± 0.27	11.10 - 17.90	7.53 - 28.70
	GHGe (kgC02eq)	4.80 ± 2.80	3.80 ± 0.12	3.05 - 5.73	2.29 - 10.00
	Agri. GHGe (kgC02eq)	2.80 ± 0.07	3.28 ± 1.82	1.97 - 4.18	1.31 - 6.61
	Nitrogen use (kg)	111.00 ± 45.60	102.00 ± 1.97	79.90 - 132.00	54.60 - 210.00
	Phosphorous use (kg)	19.30 ± 7.850	17.70 ± 0.34	13.90 - 22.80	9.34 - 36.30
51 - 64	Blue water use (L)	511.00 ± 419.00	344.0 ± 22.90	203.0 - 718.0	119.00 - 1310.00
	Cropland use (m^2)	15.10 ± 6.10	13.70 ± 0.30	10.80 - 18.00	7.59 - 27.60
	GHGe (kgC02eq)	4.70 ± 2.50	4.00 ± 0.14	3.18 - 5.66	2.23 - 8.740
	Agri. GHGe (kgC02eq)	2.95 ± 0.08	3.36 ± 1.83	1.94 - 4.28	1.25 - 7.040
	Nitrogen use (g)	19.00 ± 44.20	101.0 ± 2.42	76.5 - 131.00	55.20 - 199.00
	Phosphorous use (g)	19.00 ± 7.61	17.50 ± 0.41	13.50 - 22.70	9.50 - 34.50
65 - 88	Blue water use (L)	510.00 ± 426.00	323.00 ± 29.80	229.00 - 661.0	127.00 - 1335.00
	Cropland use (m^2)	13.80 ± 5.00	13.30 ± 0.35	10.10 - 16.20	6.53 - 23.40
	GHGe (kgC02eq)	4.90 ± 3.20	4.00 ± 0.20	3.08 - 5.75	2.11 - 10.30
	Agri. GHGe (kgC02eq)	2.79 ± 0.11	3.23 ± 1.89	1.86 - 4.14	1.15 - 6.71
	Nitrogen use (g)	99.30 ± 36.80	94.70 ± 2.58	70.80 - 116.00	46.80 - 171.00
	Phosphorous use (g)	17.30 ± 6.37	16.60 ± 0.45	12.60 - 20.30	8.21 - 29.80

Abbreviations: L = litres. kgC02 eq. = kilograms carbon dioxide equivalent. m^2 = metres squared. g = grams. Agri. Greenhouse Gas Emissions (kgC02eq) = emissions assigned to agricultural commodities. Greenhouse Gas Emissions (kgC02eq) = emissions assigned to foods as consumed from LCA database and includes emissions related to processing, transport etc.

Table S3cont. Environmental impact of average reported diets by age group for males.

Age group	Environmental factor	Males			
		Mean ± SD	Median ± SEM	IQR	95% CI
5 - 8	Blue water use (L)	95.20 ± 35.50	86.30 ± 3.10	72.10 - 113.00	53.40 - 149.00
	GHGe (kgC02eq)	2.92 ± 0.83	2.77 ± 0.07	2.34 - 3.48	1.78 - 4.41
9 - 12	Blue water use (L)	117.00 ± 52.50	108.00 ± 4.90	83.10 - 139.00	54.40 - 204.00
	GHGe (kgC02eq)	3.60 ± 1.03	3.47 ± 0.09	2.91 - 4.22	2.12 - 5.58
13 - 15	Blue water use (L)	123.00 ± 75.30	106.00 ± 8.70	71.10 - 174.00	33.00 - 246.00
	GHGe (kgC02eq)	2.89 ± 1.17	2.72 ± 0.14	2.14 - 3.23	1.39 - 4.75
16 - 18	Blue water use (L)	212.00 ± 214.00	162.00 ± 29.40	97.70 - 266.00	48.80 - 469.00
	GHGe (kgC02eq)	3.62 ± 1.43	3.33 ± 0.19	2.60 - 4.35	1.80 - 6.05
18 - 35	Blue water use (L)	406.00 ± 496.00	248.00 ± 19.90	144.00 - 507.00	72.80 - 1183.00
	Cropland use (m2)	22.40 ± 13.10	18.04 ± 0.50	13.40 - 27.10	8.85 - 50.00
	GHGe (kgC02eq)	9.20 ± 6.90	6.47 ± 0.30	4.45 - 11.50	3.11- 24.50
	Agri. GHGe (kgC02eq)	4.02 ± 0.09	4.75 ± 2.77	2.74 - 6.10	1.78 - 10.10
	Nitrogen use (kg)	157.00 ± 85.00	134.00 ± 3.41	96.30 - 194.00	63.20 - 337.00
	Phosphorous use (kg)	27.20 ± 14.60	23.40 ± 0.58	16.80 - 33.60	11.30 - 58.30
36 - 50	Blue water use (L)	641.00 ± 603.00	402.00 ± 28.60	263.00 - 794.0	128.0 - 1955.00
	Cropland use (m^2)	18.30 ± 9.32	15.90 ± 0.44	12.20 - 21.10	8.50 - 39.20
	GHGe (kgC02eq)	7.50 ± 5.17	5.60 ± 0.20	4.10 - 8.86	2.90 - 17.80
	Agri. GHGe (kgC02eq)	4.11 ± 0.11	4.81 ± 2.84	2.77 - 6.15	1.68 - 9.86
	Nitrogen use (kg)	129.00 ± 61.50	114.00 ± 2.91	88.80 - 153.00	59.80 - 259.00
	Phosphorous use (kg)	22.50 ± 10.50	20.00 ± 0.49	15.50 - 26.40	10.70 - 44.90
51 - 64	Blue water use (L)	573.00 ± 459.00	395.00 ± 266.00	262.00 - 712.00	166.00 - 1562.00
	Cropland use (m^2)	17.30 ± 7.00	15.70 ± 0.40	12.70 - 19.60	9.30 - 31.30
	GHGe (kgC02eq)	7.17 ± 4.36	5.55 ± 0.25	4.00 - 9.23	2.90 - 16.00
	Agri. GHGe (kgC02eq)	3.98 ± 0.12	4.61 ± 2.46	2.65 - 5.91	1.79 - 9.45
	Nitrogen use (g)	122.00 ± 45.80	112.00 ± 2.65	90.30 - 142.00	66.40 - 215.00
	Phosphorous use (g)	21.20 ± 7.83	19.7 ± 0.45	15.80 - 24.80	11.80 - 37.10
65 - 88	Blue water use (L)	511.00 ± 443.00	350.00 ± 34.50	248.00 - 640.00	179.00 - 1183.00
	Cropland use (m^2)	16.30 ± 7.68	14.40 ± 0.59	11.40 - 18.80	8.00 - 33.50
	GHGe (kgC02eq)	6.80 ± 4.42	5.15 ± 0.34	3.90 - 7.90	2.10 - 16.00
	Agri. GHGe (kgC02eq)	3.62 ± 0.16	4.27 ± 2.53	2.41 - 5.57	1.53 - 8.72
	Nitrogen use (g)	115.00 ± 53.20	101.00 ± 4.14	80.30 - 136.00	59.00 - 224.00
	Phosphorous use (g)	20.20 ± 9.15	17.60 ± 0.71	14.10 - 23.60	10.30 - 38.80

Abbreviations: L = litres. kgC02 eq. = kilograms carbon dioxide equivalent. m^2 = metres squared. g = grams. Agri. Greenhouse Gas Emissions (kgC02eq) = emissions assigned to agricultural commodities. Greenhouse Gas Emissions (kgC02eq) = emissions assigned to foods as consumed from LCA database and includes emissions related to processing, transport etc.

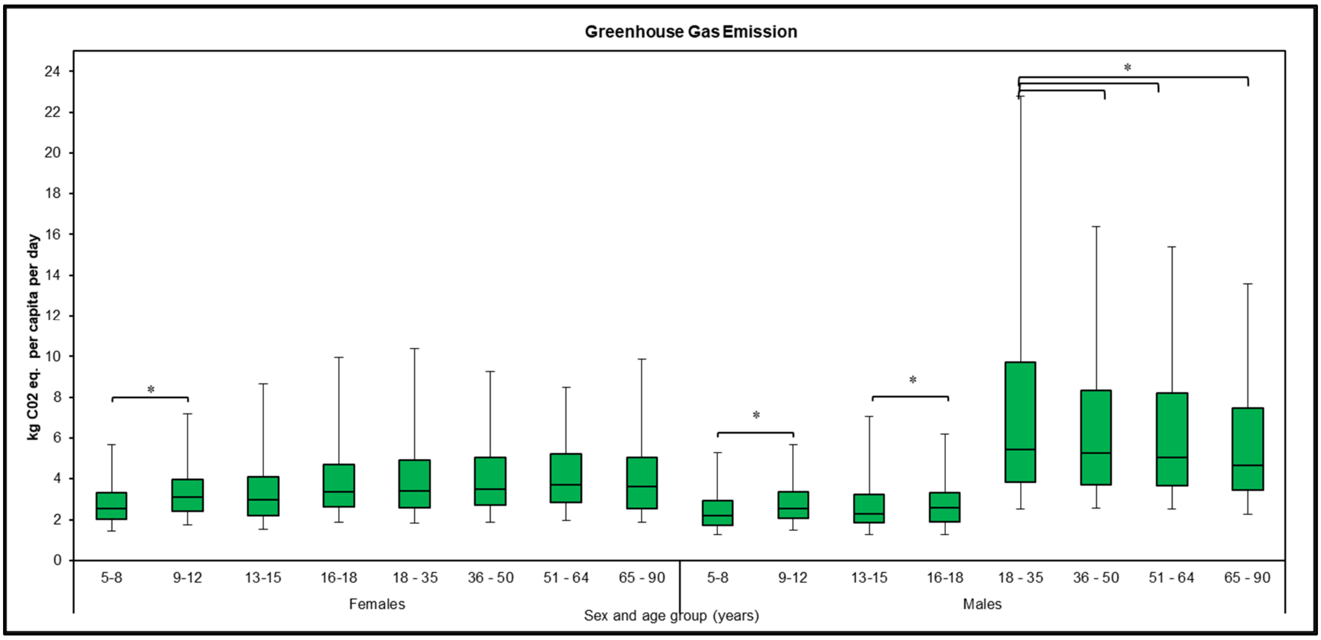

Figure S1. GHGe from daily reported diets in the food consumption surveys across population groups**.** Values shown as median, interquartile range (IQR) with 95% confidence intervals shown by whiskers. *Denotes significance using ANOVA with Bonferroni corrections for multiple comparisons (P<0.05). Includes diets reported for children *n* 2,375; teenagers *n* 798; adults *n* 4,575.

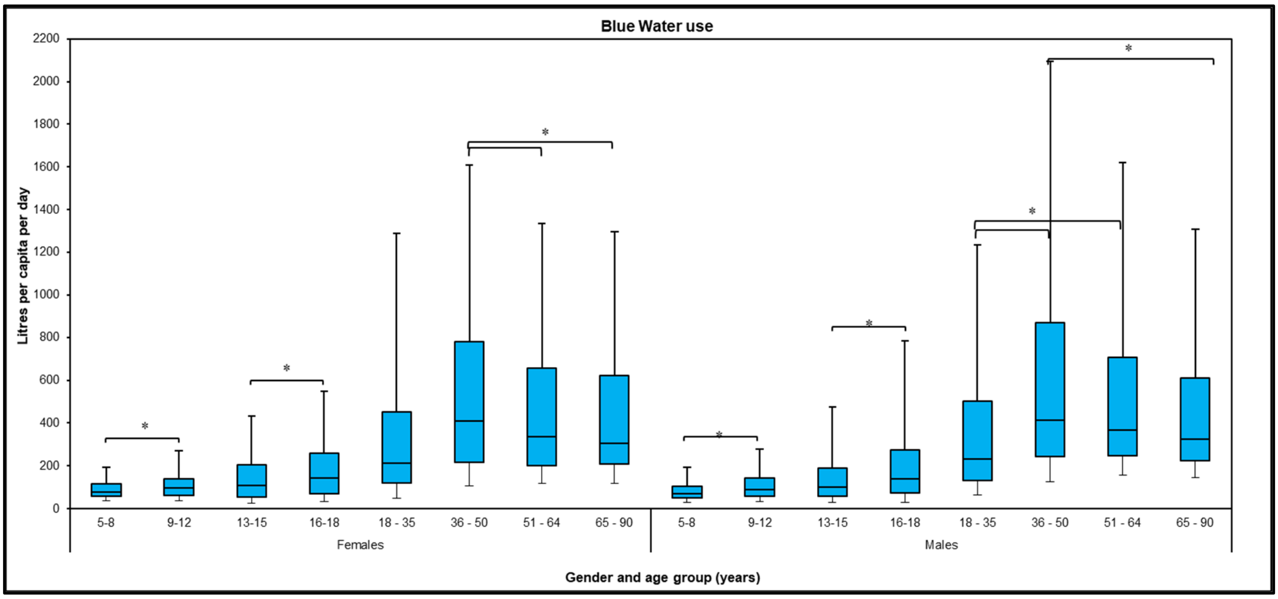

Figure S2. Blue water use from daily reported diets in the food consumption surveys across population groups. Values shown as median, interquartile range (IQR) with 95% confidence intervals shown by whiskers. * Denotes significance with at least one other age group using ANOVA with Bonferroni corrections for multiple comparisons. Includes diets reported for children *n* 2,375; teenagers *n* 798; adults *n* 4,575.

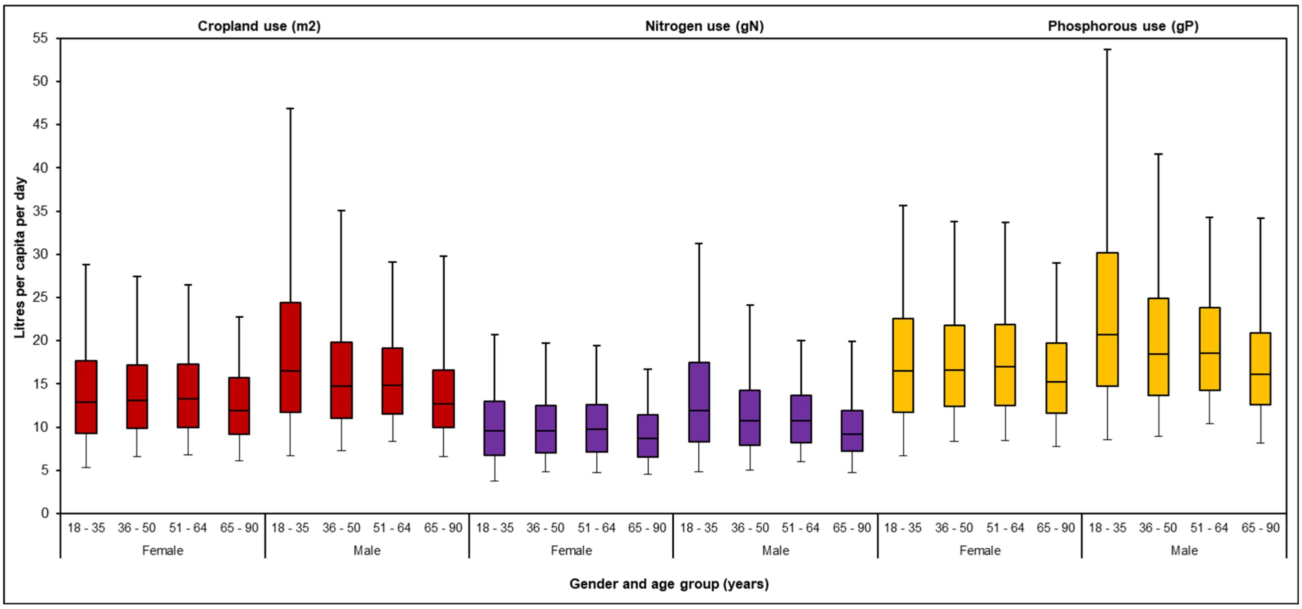

Figure S3. Cropland, nitrogen, and phosphorus use for from daily reported diets for adults. Values shown as median, interquartile range (IQR) with 95% confidence intervals shown by whiskers. * Denotes significance with at least one other age group using ANOVA with Bonferroni corrections for multiple comparisons. Includes diets reported for children n 2,375; teenagers n 798; adults n 4,575.
